# MicroRNA Let-7b-5p Induces Electroacupuncture Tolerance by Downregulating the MKP-1 Gene in Rats Subjected to CFA-induced Inflammatory Nociception

**DOI:** 10.1007/s12031-020-01527-6

**Published:** 2020-04-02

**Authors:** Qiulin Zhang, Mahmoud M. Abouelfetouh, Shuhuai Chen, Meng Li, Mingxing Ding, Yi Ding

**Affiliations:** College of Veterinary Medicine, Huazhong Agricultural University, No.1, Shizishan Street, Hongshan District, Wuhan, 430070 Hubei People’s Republic of China

**Keywords:** EA tolerance, p38MAPK, Let-7b-5p, MicroRNA, MKP-1

## Abstract

Electroacupuncture (EA) treatment has proved to significantly decrease nociception in inflammatory nociception model by suppressing the phosphorylation of p38 mitogen-activated protein kinase (p38MAPK). However, repeated EA treatment results in gradual attenuation of its analgesic effects, which was defined as “EA tolerance.” Recent studies have shown that let-7b-5p microRNA (miRNA) contributes to the EA tolerance. The present study aimed to explore the function of let-7b-5p in p38MAPK pathway and the development of EA tolerance in the inflammatory nociception. Dual luciferase reporter gene experiments were used in cortical neurons to determine the target gene locus of let-7b-5p. The threshold of nociception was assessed by tail flick latency (TFL) and paw withdrawal threshold (PWT). Western blots were used to measure the expression of mitogen-activated protein kinase phosphatase 1 (MKP-1) and phosphorylation level of p38MAPK after intracerebroventricular (ICV) injections of let-7b-5p agomir, antagomir, and controls. In vitro dual luciferase experiments demonstrated that the MKP-1-3′ untranslated region (UTR) is a target of let-7b-5p. In vivo experiment, rat with repeated EA treatment exhibits gradual decrease in TFL and PWT, which showed formation of EA tolerance. This trend was delayed after IVC injection of let-7b-5p antagomir and facilitated after IVC injection of let-7b-5p agomir. The protein levels of MKP-1 in the EA+let-7b-5p antagomir group were significantly higher than in the EA + let-7b-5p agomir group. However, P-p38MAPK in the EA+let-7b-5p antagomir group was significantly lower than in the EA+let-7b-5p agomir group. By upregulating the p38MAPK pathway through the inactivation of the MKP-1 gene, let-7b-5p contributes to EA tolerance in complete Freund’s adjuvant (CFA)-induced inflammatory nociception rats. Our work revealed the mechanism of EA tolerance and indicated that let-7b-5p could be targeted to improve the long-term effects of EA.

**Electronic supplementary material:**

The online version of this article (10.1007/s12031-020-01527-6) contains supplementary material, which is available to authorized users.

## Introduction

Acupuncture has been used as a medical technique in Asian countries for a long time. Likewise, it was recognized by the World Health Organization as an effective treatment in 2003 (Zhu 2002). Since then, researches of controlled clinical trial and experiment on acupuncture have been expanded. Electroacupuncture (EA) is stemmed from traditional manual acupuncture and now widely used for its better efficacy, objectivity, and controllability than manual acupuncture. EA has been applied as a preventative method for various disorders and could also be used to treat diseases. Among its numerous applications, EA-induced analgesia has gained the most interest and can be used as a complementary and alternative way to manage chronic pain when long-term medication use is undesirable. EA has nearly no side effects and can be used for prolonged durations. However, individuals showed the decrease or even loss of EA-induced analgesic effect after repeated or prolonged application of acupuncture, which is defined as “acupuncture tolerance” (Han et al. 1981). Exploring the mechanism of this phenomenon would facilitate the use of EA and improve the wellness of patients with chronic nociception. Our previous study has demonstrated that let-7b-5p microRNA (miRNA) contributes to the formation of EA tolerance; forty-nine miRNAs are differently expressed in the rat hypothalamus between the electroacupuncture group and the control group, including miR-124-3p, let-7b-5p, miR-221-3p, and miR-107-3p. Among these miRNAs, let-7b-5p exhibits most significant (Cui et al. 2017). However, its specific role in the process of EA tolerance is still unclear.

miRNAs are non-protein coding RNAs containing 18-24 nucleotides that are involved in many biological processes, including cell growth, proliferation, and development. At the posttranscriptional level, miRNAs bind to the coding region or the 3′ untranslated region (3′UTR) to adjust gene expression by cleaving mRNAs or repressing translation (Fu et al. 2011; Xia et al. 2010a; Xia et al. 2010b). In neurobiology, miRNA has been reported to regulate the effects in nociception (Ying et al. 2010). Zhao et al. showed that Dicer (an enzyme that combines miRNA and facilitates miRNA targeted mRNA cleavage) deletion affects genes related to nociception, suggesting that miRNA is involved in nociception regulation. Other research has also found that miRNA expression changes in inflammatory or neuropathic nociception models (Jing et al. 2010). Several miRNAs, including miR-124a, miR-7a, miR-203, and miR-103 have been proposed to play an important role in nociceptive modulation (Alexandre et al. 2014; Atsushi et al. 2013; Kynast et al. 2013; Sun et al. 2012). Our previous research also showed that 49 miRNAs are differently expressed in the rat hypothalamus between the electroacupuncture group and the control group, including miR-124-3p, let-7b-5p, miR-221-3p, and miR-107-3p (Cui et al. 2017).

Previous research has found that the activation of p38 mitogen-activated protein kinases (p38MAPK) in the central nervous system (CNS) plays an essential role in inflammatory nociception. On the other side, mitogen-activated protein kinase phosphatase-1(MKP-1), also known as Dual specificity phosphatase 1 (*Dusp1*), is a suppressor of the p38MAPK pathway. MKP-1 is localized to the nucleus and specifically dephosphorylates the p38MAPK. EA treatment, which is used for analgesia and anti-inflammation, mainly suppresses the phosphorylation of p38MAPK in the spinal cord following inflammatory nociception (Fang et al. 2013), we therefore hypothesized that the EA tolerance resulted from the decreased EA effects on the MKP-1/p38MAPK pathway. In our previous study, we found that the let-7b-5p miRNA increases as tolerance to the EA was induced in the complete Freund’s adjuvant (CFA)-induced inflammatory nociception rats (Cui et al. 2017). In the present study, we explored the functions of let-7b-5p in EA-induced tolerance and found that let-7b-5p contributed to the formation of EA tolerance by decrease MKP-1 and upregulating the phosphorylation level of p38MAPK gene. Our work revealed the mechanism of EA tolerance and indicated that let-7b-5p could be a new target for improving the long-term effects of EA.

## Material and Method

### Isolation and Identification of Rat Cerebral Cortical Neurons

Cultures of rat cerebral cortex neurons were prepared from embryonic day 18 Sprague–Dawley (SD) rat fetuses as previously described (Cui et al. 2017). Briefly, fetuses were exteriorized from the uterus of deep anesthetized pregnant rat and then the cerebral cortex was dissected and removed from the fetal brain. The brain tissue was then washed with ice-cold phosphate buffered saline (PBS) containing 50 mg/mL penicillin-streptomycin. The cerebral cortexes were minced and incubated for 20 min at 37 °C in pre-warmed PBS containing 0.25% trypsin. The extract was then transferred to Dulbecco’s modified Eagle’s medium containing 10% horse serum and 100 U/mL penicillin-streptomycin. After suspending the cells, the neurons were plated on freshly prepared poly-L-lysine coated 24-well plates (Sigma, Ronkonkoma, NY, US). The plating medium consisted of minimal essential medium (MEM) with Earle’s salts (Invitrogen, Waltham, MA, US) and was supplemented with heat-inactivated horse serum (5%), fetal bovine serum (5%), 17 mM glucose, 400 mM glutamine, 50 U/mL penicillin, and 50 g/mL streptomycin. The neurons were cultured in a humidified incubator containing 5% CO_2_ and 95% air at 37 °C. In order to inhibit glial cell proliferation, cytosine arabinoside (6.7 mM) was added to the culture 3–4 days after plating. Semi-media exchange was carried out 4–5 days after the start of culture with Neurobasal medium (Invitrogen, Waltham, MA, US) containing B27, L-glutamine, 50 U/mL penicillin, and 50 μg/mL streptomycin. The neurons were cultured for 8–10 days according to the above conditions before they were used for in vitro experiments. The neuronal cells were identified with rabbit-anti-rat antibodies. Immunostaining was performed as reported previously (Seija et al. 2008). For primary antibodies, we used anti-microtubule-associated protein 2 (MAP 2, 1:100, Proteintech Group, Inc. Wuhan, Hubei, China), and DAPI was used to stain the nucleus (DIPA,1:2000, Proteintech Group, Inc. Wuhan, Hubei, China). Following immunostaining, cells were visualized under a fluorescence microscope (Thermo, Shanghai, China).

### Transfection

In this experiment, miRNAwalk (http://zmf.umm.uni-heidelberg.de/apps/zmf/mirwalk2/miRretsys-self.html) was used to predict potential target genes. The MKP-1 gene was selected as the target gene for subsequent experiments (Fig. 2b). The complete sequence of the MKP-1 3′UTR (National Center for Biotechnology Information reference sequence NM_053769.3) was amplified. The recognition sequences of XhoI and NotI restriction enzymes were inserted at the 5′ end and the 3′ end of the primer, respectively. Single-stranded DNA fragments containing mutated let-7b-5p seed sequence and the complementary fragment were synthesized according to a previous study that used the SOEPRC technique (Scheller et al. 2004). Four primers were used to amplify the MKP-1 gene 3′UTR wild type or mutated let-7b-5p “seed sequence” (Table 1). In order to obtain the recombinant wild-type reporter gene vector, MKP-1-3′UTR-WT and the mutant reporter gene vector MKP-1-3′UTR-Mut, the above amplified sequence was inserted into the dual luciferase reporter vector psiCHECK-2 (Promega, Madison, WI, USA). The oligonucleotides of let-7b-5p mimic (antisense: 5′-UGAGGUAGUAGGUUGUGUGGUU-3′: sense CCACACAACCUACUACCUCATT) and scrambled oligonucleotides (miR-control, antisense: 5′-UUCUCCGAACGUGUCACGUTT-3′: sense: 5′-ACGUGACACGUUCGGAGAATT-3′) were all purchased from Gene Pharma (Shanghai, China). For dual luciferase reporter assay, cortical neurons were transferred to 48-well plates in Opti-MEM® I Medium and co-transfected with 0.8 μg pischeck-2 reporter gene vector (MKP-1-3′UTR-WT and MKP-1-3′UTR-Mut) and 20 pM of the let-7b-5p mimic, or miR-control according to the specification via lipofectamine2000™ (Invitrogen, Carlsbad, CA, USA). As a blank control, cortical neurons were transfected with the pischeck-2 vector. After transfection and culture for 6 h, the new medium was replaced. Cells were lysed 30 h after transfection and analyzed using the dual luciferase reporter system (Promega, Madison, WI, USA). All experiments were performed in triplicates and independently.

**Table 1:** The primers of amplification MKP-1 gene 3′UTR

Gene	Sequence
MKP-1-3UTR-xhoI-F	TCAGctcgagCTTCGTGCTTGACATACCTACCAG
MKP-1-3UTR-NotI-R	AATTgcggccgcCCAACACTGGCTTCGTCCATCAG
MKP-1-F1	ATTGTCTACTCATAGAAGAACGAAACTTTTTGTGTTTGCGTACTGTACTA
MKP-1-R1	TAGTACAGTACGCAAACACAAAAAGTTTCGTTCTTCTATGAGTAGACAAT
Stable negative control	5′ UUCUCCGAACGUGUCACGUTT 3′
	3′ ACGUGACACGUUCGGAGAATT 5′
Agomir	5′UGAGGUAGUAGGUUGUGUGGUU3′
	3′CCACACAACCAUCUACCUCAUU5′
Antagomir	5′ AACCACACAACCUACUACCUCA3′
let-7b-cDNA primer	GTCGTATCCAGTGCGTGTCGTGGAGTCGGCAATTGCACTGG ATACGACAACCACAC
let-7b-f	GTAGGTTGTGTGGTTGTCGT
let-7b-r	CCAGTGCGTGTCGTGG

### Model Establishment

Female SD rats weighing 280 ± 20 g were provided by the Hubei Provincial Center for Disease Control and Prevention. The rats were allowed free access to food and water. About 6–7 rats were housed in each cage. All rats were acclimatized to the environment for a week to reduce stress before experiments.

The method for ICV injections had been reported previously (Deng et al. 2013). In short, after anesthesia (sodium pentobarbital 40 mg/kg), rats were fixed on a stereotactic apparatus. The skull was then surgically exposed and a small hole was micro-drilled on the skull at the following coordinates: 1.5 mm lateral to and 0.8 mm posterior to the bregma. Then the microtubule was inserted into the skull below the skull surface and fixed at 4.0 mm. All rats were recovered for at least 7 days post-surgery and adapted to the fixed cylinder for 3 days (30 min one time) before the formal experiment. The let-7b-5p agomir, antagomir, antagomir control, and agomir control (0.2 nmoL/μL each, added to 5 μL in saline, purchased from Gene Pharma, Shanghai, China) were injected at a rate of 1 μL/10 min through the cannula to the lateral ventricles (Cui et al. 2017). The base sequences of the ICV injection drugs are listed in Table 1. The doses of the drugs used were determined by a previous study (Cui et al. 2017; Deng et al. 2013). The rats were divided into 4 groups: (1) EA+Agomir, (2) EA+ Antagomir, (3) EA+ Agomir control, and (4) EA+Antagomir control. There were 18 rats in each group. The experiment lasted for 8 days.

The inflammatory nociception model was established by subcutaneously injecting 0.1 mL CFA (Sigma, USA) into the right hind paw of all rats 2 days before EA stimulation; ICV injections of agomir, antagomir, and controls were performed 1 day before EA stimulation.

### Electroacupuncture Stimulation

The rats in the EA group were given EA stimulation at a fixed time of the day (9:00 am) using an improved method reported by Min-feng et al. (Minfeng and Jisheng 1979). The hind legs and tail were exposed and the rats were placed into a specially designed fixed cylinder holder. The ambient temperature was controlled at 21~23 °C. A stainless steel needle (0.30 mm in diameter, 13 mm in length) was inserted into the acupuncture point on both sides of the leg that had been sterilized: “Zusanli” acupoint (ST36, a notch 4 mm lateral to the anterior tuber point of the tibia, 6–7 mm depth) and the “Sanyinjiao” acupoint (SP6, 3 mm proximal to the middle malleolus at the posterior border of the tibia, 4–5 mm depth). The needles were connected to WQ-6F Electronic Acupuncto-scope (Beijing Xindonghua Electronic Instrument Co., Ltd., Beijing, China). The stimuli were set as square waves, 2 Hz in frequency, with an amplitude of 3 mA, 30 min/day for 7 days (Cui et al. 2017). The rats in the sham group were inserted with needle without giving any EA stimulation.

### Measurement of Nociceptive Threshold

Nociceptive threshold was assessed by the tail flick latency (TFL) response and paw withdrawal threshold (PWT). TFL test used radiant heat stimuli emitting from the YLS-12A Tail Flick Analgesia Instrument (ZS Dichuang Science and Technology Development Co., Ltd., Beijing, China). Radiant heat from the instrument was used to stimulate the proximal third of the tail and the TFL was then observed. The baseline incubation period in the range of 4–6 s was set by adjusting the intensity of the thermal stimulus. In total, 15 s was the cutoff limit in order to prevent tissue damage. The baseline TFL was determined as the average of 3 consecutive measurements taken at 5 min intervals prior to EA stimulation. TFL was measured every 10 min during EA stimulation for 30 min. The following formula was used to calculate the percentage increase of TFL. The EA-induced antinociception was defined from the 3 assessments: TFL (%) = (latency after EA − latency before TFL)/latency before TFL × 100%.

The paw withdrawal threshold (PWT) was also used to assess the inflammatory pain. Rats were first acclimatized in individual plastic enclosures on a metal mesh stand for 10 min before testing. The mechanical stimulus was delivered to the plantar surface of right hind paw from below the floor of the plastic enclosures by an electronic von Frey aesthesiometer (ZS-Dichuang Science and Technology Development Co., Ltd., Beijing, China). A force transduction fitted with a 0.5-mm diameter polypropylene rigid tip was applied perpendicularly against the hind paw with an ever increasing force from 0 to 50 g during a 20-s period. When the rat withdrew its hind paw, the mechanical stimulus was automatically stopped, and the force was recorded as PWT by the aesthesiometer. The procedure was repeated three times with 5 min interval. The PWT was measured before and after EA stimulation three times with an interval of 5 min each time. The percentage change in PWT was calculated using the following formula: PWT% = (PWT after EA−PWT before EA)/PWT before EA × 100%.

### Western Blots of MKP-1 and p38MAPK

The rats were given EA once daily for seven consecutive days. At days 1, 4, and 7, 2 h after EA stimulation, 6 rats in each group were euthanized and the brains were rapidly dissected and the hypothalamus was isolated on an ice cube treated with diethyl carbonate (DEPC) water as previously reported (Gao et al. 2007; Wen 2012). The isolated hypothalamus was immediately stored in liquid nitrogen for RNA isolation and protein extraction.

Manually homogenized samples were stored in liquid nitrogen and added to radioimmunoprecipitation assay (RIPA) buffer (1% sodium deoxycholate, leupeptin, sodium fluoride, 0.1% sodium dodecyl sulfate (SDS), sodium orthovanadate, ethylenediaminetetraacetic acid (EDTA), 150 mM NaCl, 1% 50 mM Tris, pH 7.4, and Triton X-100) containing phenylmethylsulfonyl fluoride (PMSF), the protease inhibitors, and a phosphatase inhibitor. The homogenate was allowed to stand on ice for 30 min and centrifuged (15,000 relative centrifugal force, 4 °C, 15 min) to obtain the supernatant. Protein concentration was determined by the bicinchoninic acid (BCA) protein assay kit. Proteins were separated in a 10% SDS-PAGE gel and transferred to polyvinylidene fluoride (PVDF) membrane by electrophoresis (GE Healthcare, Waukesha, WI, USA). A total of 5% low-fat milk in tris-buffered saline with Tween 20 (TBST) was used to block the membranes for 2 h at 37 °C Rabbit anti-rat β-actin and anti-rat P-p38MAPK (1:1000, CST, Danvers, MA, USA) was used as an internal control. Rabbit anti-MKP1 (1:100, Boster, Wuhan, China) and rabbit anti-rat p-p38 MAPK (1:1000, CST, Danvers, MA, USA) were used as primary antibodies, and horseradish peroxidase (HRP)-conjugated goat anti-rabbit IgG was used as the secondary antibody (1:5000). The membranes were developed using the enhanced chemiluminescence (ECL) kit (Pierce, Rockford, IL, USA) and the signals were captured with Image Quant LAS 4000 (GE Healthcare, Waukesha, WI, USA). Image Quant TL7.0 Analysis Software (GE Healthcare, Waukesha, WI, USA) was used to analyze the scanned images.

### RNA Isolation and Quantitative Polymerase Chain Reaction

Hypothalamus samples were collected in the same way that the tissue was collected in for western blots. Total RNAs were isolated with the Trizol reagent (Takara, Shiga, Japan). Using the reverse transcription kit (Transgen, Beijing, China), RNA was reverse transcribed into cDNA. The levels of miRNA and mRNA were quantified using quantitative polymerase chain reactions (qPCRs). Specifically, samples were incubated at 95 °C for 10 min, followed by 40 cycles at 95 °C for 15 s, and 60 °C for 1 min. β-actin and GAPDH were used as internal controls. Stem loop quantitative reverse transcriptase qPCR (qRT-PCR) assays were used for the detection of miRNAs as described previously. Primer sequences are listed in Table 1.

### Statistical Analysis

SPSS software version 21.0 was used for statistical analyses (SPSS Inc., Chicago, IL, USA). All data were presented as mean ± SD. Variables were analyzed using one-way ANOVAs followed by Bonferroni post-hoc tests where appropriate. *P* < 0.05 was considered statistically significant.

## Results

### Let-7b-5p miRNA Directly Targets and Negatively Regulates MKP-1

**Fig. 1:**
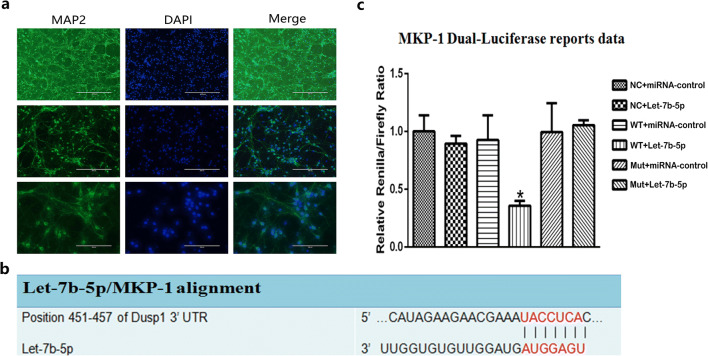
Let-7b-5p directly targets and negatively regulates MKP-1. **a** Rat cortical neurons were identified with antibodies to microtubule-associated proteins (MAP 2), which is specific to neurons. Scale bar represents 400 μm in the pictures at the first line, 200 μm in the pictures at the second line, and 100 μm in the pictures at the third line. **b** Predictive analysis of possible binding sites of the MKP-1 gene. **c** The Renilla luciferase activity in the transfected cortical neurons. Data were presented as mean ± SD (*n* = 3): There is significant difference in WT + let-7b-5p group when compared with other groups (*p* < 0.05). * Significant difference between groups. WT, wild type; Mut, mutant; Let-7b-5p, Let-7b-5p mimic; 3′UTR, 3′-untraslated regions; DAP1, death-associated protein 1

Our previous work found that let-7b-5p play an important role in EA tolerance; however, the target of this miRNA and its regulatory mechanism is remaining elusive. Therefore, using the miRNAWalk 3.0 online database, (http://zmf.umm.uni-heidelberg.de/apps/zmf/mirwalk2/), we found that the seed regions of let-7b-5p matched sites in the 3′UTR of MKP-1 (Fig. 1b), the full list of let-7b-5p targets were shown in Supplementary Table 1. A 223 bp, rat MKP-1 3′-UTR DNA fragment, containing the putative let-7b-5p interaction sequence, was constructed and cloned into the psiCHECK-2 vector (psiCHECK-MKP-1-3′UTR) as WT plasmid. A deletion mutant of this 3′UTR fragment that lacked the putative let-7b-5p target sequences was also constructed (psiCHECK-MKP-1-3′UTRmut) as Mut plasmid. A psiCHECK-2 vector without any change was used as NC plasmid. These plasmids were transfected into the cortex neural cells with let-7b-5p mimics and miR-control, which were purchased from Gene Pharma (Shanghai, China). Transfection efficacy was normalized to the internal control of firefly luciferase activity. Immunofluorescent staining with anti-MAP 2 was used to identify neuronal cells, and the cell purity was over 90% (Fig. 1a). As presented in Fig. 1 c, the luciferase assay was applied to validate if let-7b-5p binds directly to the 3′UTR of MKP-1. The relative renilla/firefly ratio in the WT plasmid and let-7b-5p mimics transfected cortex neural cells was only 40% of those transfected with NC plasmid and let-7b-5p mimics (Fig. 1c). There is no significance detected between groups with NC plasmid+let-7b-5p mimics transfection and groups with NC plasmid +miRNA control, WT plasmid+miRNA-control, Mut plasmid+miRNA-control, and Mut plasmid+let-7b-5p mimic transfection. These data suggested that MKP-1-3′UTR is a target gene of let-7b-5p, and its expression is negatively correlated with let-7b-5p expression during the induction of EA tolerance.

### Let-7b-5p Expression Increased as EA Tolerance Was Induced in CFA-induced Inflammatory Nociception Rats

**Fig. 2:**
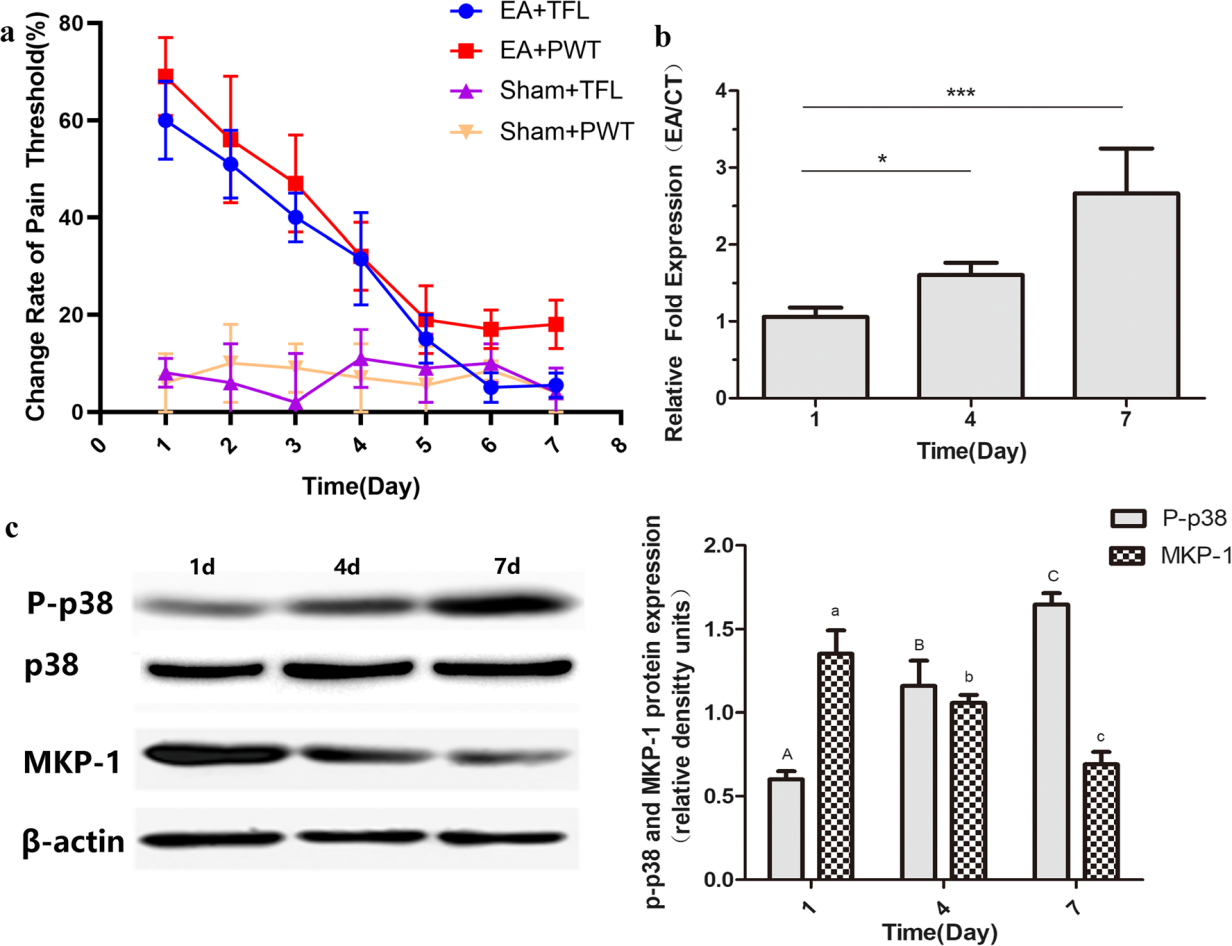
The expression level of let-7b-5p in the hypothalamus tissue. **a** Changes in nociceptive threshold after repeated EA treatments (mean ± SD, %; *n* = 6). The change of TFL and PWT is decreased from day 1 to 7. **b** Relative expression level of let-7b-5p in EA tolerance group at days 1, 4, and 7 (mean ± SD, *n* = 6). The expression level of let-7b-5p at days 4 and 7 is significantly higher than day 1. * *p* < 0.05, *** *p* < 0.0001. The relative fold expression (EA/CT) was drawn by comparing data from EA group with EA sham group. **c** The phosphorylation levels of P-p38MAPK and expression level of MKP-1 in EA tolerance group at days 1, 4, and 7. The P-p38MAPK gradually increased from day 1 to day 7, and the expression level of MKP-1 gradually decreased from day 1 to day 7. The values with different letters differ significantly (mean ± SD, *n* = 6, *p* < 0.05)

The in vitro experiment showed that let-7b-5p could target on MKP-1 gene. As MKP-1 is the suppressor gene of p38MAPK, we hypothesized that let-7b-5p upregulate phosphorylation of p38MAPK through downregulating MKP-1. Therefore, we further use CFA-induced inflammatory nociception rats to explore the relationship between let-7b-5p and MKP-1/p38MAPK pathway. The rats were given EA repeatedly every day for 7 days. The TFL and PWT were measured at each day (Fig. 2a). The expression of let-7b-5p were examined at days 1, 4, and 7 in the hypothalamus; the result showed that expression level of let-7b-5p increased during the formation of EA tolerance (Fig. 2b). The levels of MKP-1 and P-p38MAPK were also measured using western blots. As shown in Fig. 2 c, the phosphorylation level of p38MAPK on days 4 and 7, when compared with day 1, increased as the EA tolerance was induced (*p* < 0.0001; *p* = 0.0029). Meanwhile, the expression level of MKP-1 was significantly decreased on days 4 and 7 when compared with day 1 (*p* = 0.0005, 0.0103 on days 4 and 7, respectively).

### Changes in Nociception Threshold After IVC Injections of Let-7b-5p Agomir, Antagomir, and Controls

**Fig. 3:**
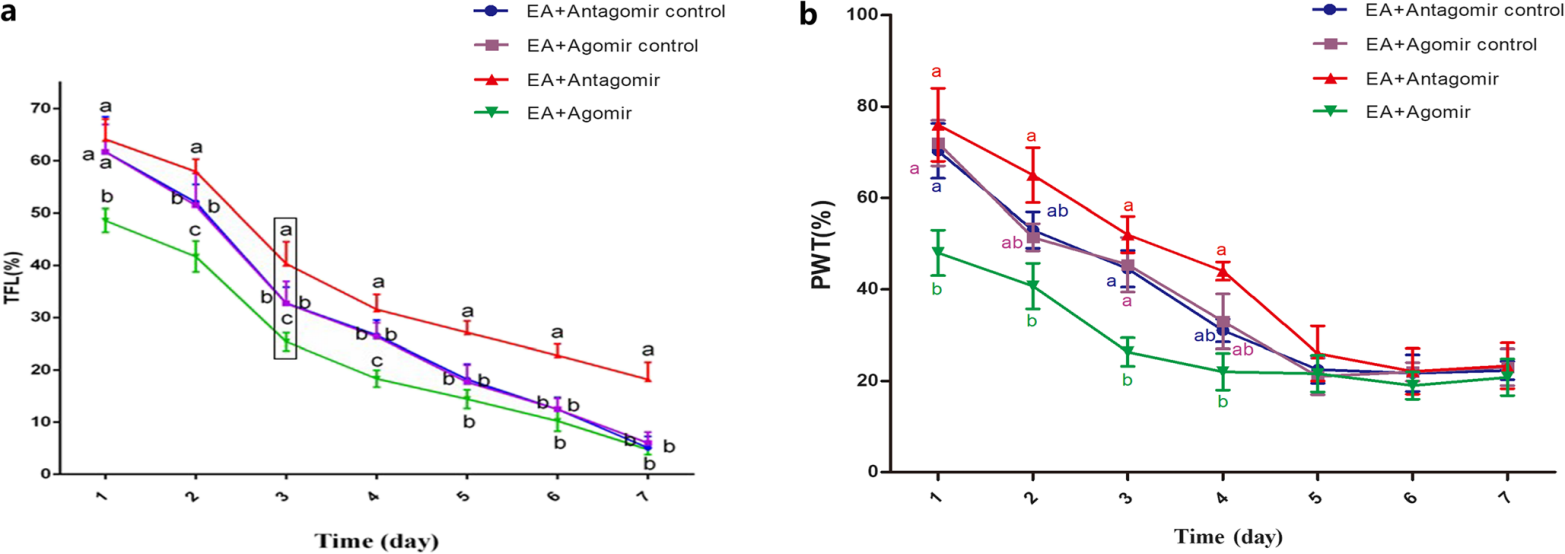
Changes in nociception threshold after IVC injections of let-7b-5p agomir and antagomir. **a** Changes in tail flick latency after repeated EA treatments. The change of TFL is significantly higher in EA + Antagomir and significantly lower in EA + Agomir when compared with EA + control groups. The significance of differences (*p* < 0.05) was marked with different letters (a–c) (mean ± SD, %; *n* = 6) **b** Changes in paw withdrawal threshold after repeated EA treatments. The change of PWT is higher in EA + Antagomir than in EA + control group but there is no statistical significance. The EA + Agomir is significantly lower than the EA + control group. The significance of differences (*p* < 0.05) was marked with different letters (a–c) (mean ± SD, %; *n* = 6)

In order to test whether there is a causal relationship between let-7b-5p and EA tolerance, we injected let-7b-5p agomir, antagomir, and controls intraventricularly. Changes in TFL were observed over 7 consecutive days (Fig. 3a). There were no significant differences in changes in TFL between the EA+Agomir control and the EA+Antagomir control group. The change in TFL in the EA+Antagomir group was significantly higher than in the EA+Antagomir control group from days 2–7 (*p* = 0.002, *p* < 0.0001, *p* = 0.0002, and *p* < 0.0001 on days 5–7) and higher than EA+Agomir group from days 1–7 (*p* < 0.0001 on days 1–7). The percent change in TFL in the EA+Agomir group was lower than in the EA+Agomir control from days 1–4 (*p* < 0.0001 on days 1–4), but no differences were observed from days 5 to 7. Changes in PWT were also observed over 7 consecutive days (Fig. 3b). The changes in PWT were higher in EA + Antagomir groups when compared with the EA + Antagomir control groups. However, there is no statistical significance. The change in PWT in the EA + Antagomir group was significantly higher than in the EA + Agomir group from days 1–4 (*p* = 0.0253, 0.0064, 0.0034, 0.0003 on days 1, 2, 3, and 4, respectively). In 1, 3, and 4 days, the change in PWT in the EA + Agomir group was significantly lower than in the EA + Agomir control group (*p* = 0.0432, 0.0136, 0.0125 on days 1, 3, and 4, respectively). From day 5, there were no significant differences between the 4 groups. These results indicated that let-7b-5p contributes to chronic EA tolerance in the CFA-induced inflammatory nociception model.

### Expression Level of MKP-1 and Phosphorylation Level of p38MAPK Following Injections of Let-7b-5p Agomir and Antagomir

**Fig. 4:**
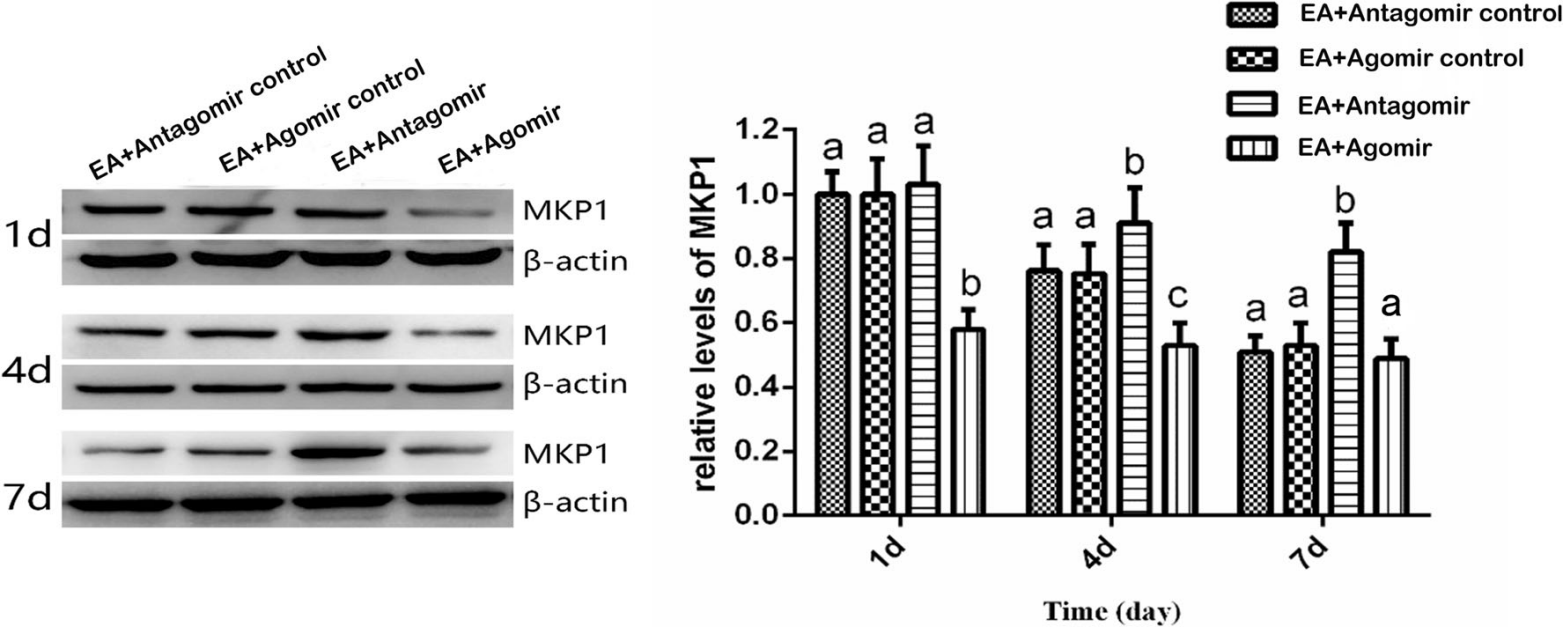
The protein expressions of MKP-1 after different treatment in EA tolerance rats (mean ± SD, *n* = 6). The expression level of MKP-1 is higher in the EA + Antagomir group than in the EA + Antagomir control in days 4 and 7. The EA + Agomir is lower than the EA + Agomir control group in days 1 and 4. The significance of differences (*p* < 0.05) was marked with different letters (a–c) and calculated by a *t* test

**Fig. 5:**
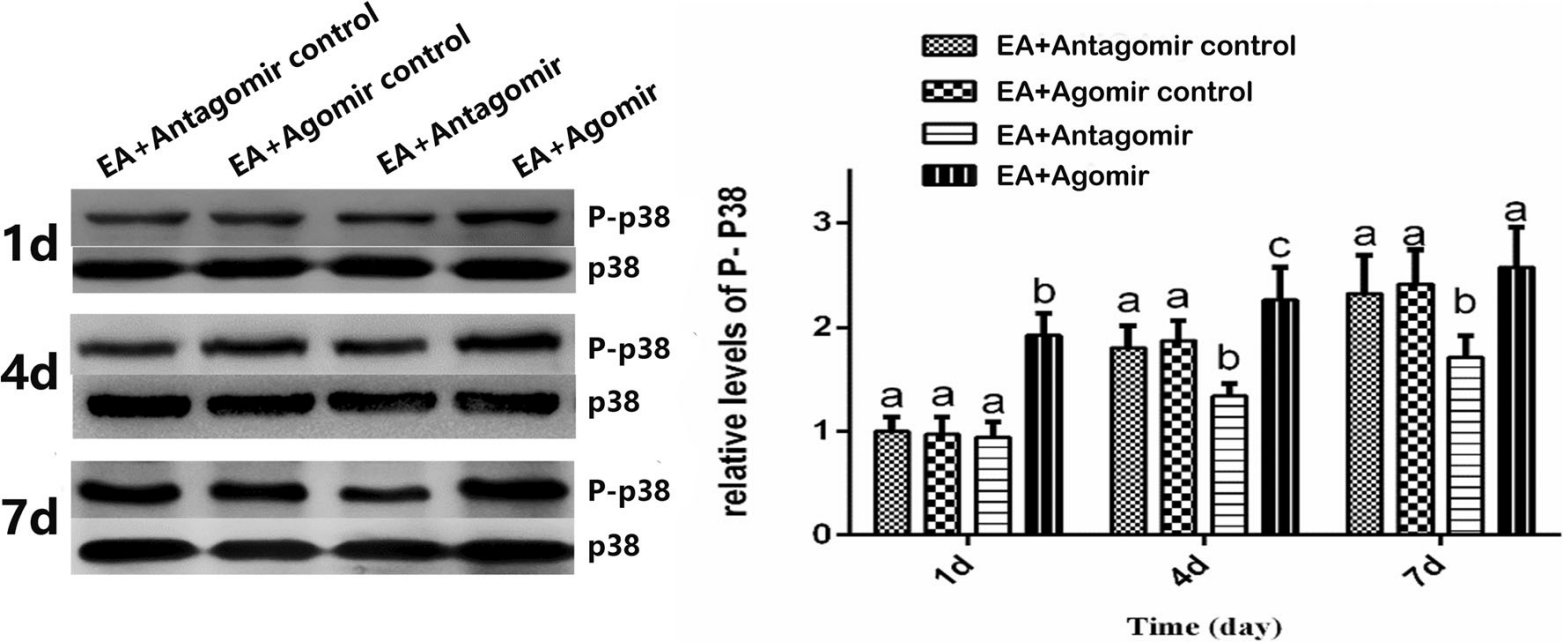
The protein level of P-p38MAPK after different treatments in EA tolerance rats (mean ± SD, *n* = 6). The protein level of P-p38MAPK is lower in the EA + Antagomir than in the EA + Antagomir control group in days 4 and 7. The protein level of P-p38MAPK is significantly higher in the EA + Agomir than in the EA + Agomir control group in days 1 and 4. The significance of differences (*p* < 0.05) was marked with different letters (a–c) and calculated by a *t* test

To verify the causal relationship between let-7b-5p and MKP-1/P-p38MAPK pathway, the let-7b-5p agomir and antagomir were injected intraventricularly. Meanwhile, the expression level of MKP-1 and the phosphorylation level of p38MAPK were determined at days 1, 4, and 7 after the EA. The expression of β-actin and p38MAPK were used as internal control. As shown in Fig. 4, compared with the EA + Agomir control, the protein level of MKP-1 in the EA + Agomir group was significantly lower on days 1 (*p* = 0.0322) and 4 (*p* = 0.0156). There was a significant higher protein level of MKP-1 in the EA + Antagomir group on days 4 (*p* = 0.0156) and *7* (*p* = 0.0375) compared with the EA + Antagomir control group. Meanwhile, the expression of MKP-1 gradually decreased over the time course of EA treatment. As shown in Fig. 5, the level of P-p38MAPK on days 1,4, and 7 was significantly higher in the EA + Agomir group than in the EA + Agomir control group on days 1 and 4 (*p* < 0.0001; *p* = 0.0052). Compared with the EA + Antagomir control, the protein level of P-p38MAPK in the EA + Antagomir group was significantly lower on days 4 (*p* = 0.0022) and 7 (*p* = 0.0005) after EA, and as time progressed, the protein level of P-p38MAPK gradually increased in all group. These findings showed that let-7b-5p upregulating the p38MAPK pathway through the inactivation of the MKP-1 gene contributes to the formation of EA tolerance.

## Discussion

Our previous research showed that the expression of let-7b-5p was significantly increased in the hypothalamus during the formation of EA tolerance via a deep sequencing technique (Cui et al. 2017). However, the mechanism underlying let-7b-5p mediated EA tolerance has not been fully elucidated. In the present study, we found that let-7b-5p could directly target the MKP-1 gene and contribute to the formation of EA tolerance by affecting the MKP-1/p38MAPK pathway.

In our study, the suppressive effect of let-7b-5p on target gene, MKP-1, was validated by Duel-Luciferase reporter gene system in the cerebral cortex neural cells, instead of hypothalamus neural cells. This is mainly because the hypothalamus of fetus rats is too small to isolate. The reporter systems have been used in a variety of cell types. The type of cells used in the experiment should not affect the target of miRNA (Nie et al. 2017; Yan et al. 2017; Zhang et al. 2017). The effect of let-7b-5p on EA tolerance was investigated in the CFA-induced inflammatory nociception rat model in this study. As the rats in the model were given EA treatments repetitively, changes in TFL gradually decreased, which indicated the formation of EA tolerance. We chose to use the CFA-induced inflammatory nociception model over models under physiological status because this model is more clinically relevant. Interestingly, EA tolerance could be induced in the CFA-induced inflammatory nociception model in a much shorter period of time. Cheng et al. (2008) found that changes in TFL in the CFA-induced inflammatory nociception rat model was 65.2 ± 5.0% at day 1 and reduced to 32.4 ± 2.1% by day 3. However, changes in TFL of rats in the physiological status was 49.9 ± 5.7% at day 1 and reduced to 31.6 ± 3.7% by day 4 (Cheng et al. 2008). The P-p38MAPK is reported to be increase in the CFA-induced inflammatory nociception model instead of physiological status (Gao et al. 2017). Meanwhile, P-p38MAPK is also increased during the formation of EA tolerance based on our result in the present study. It is possible that the synergistic activation of the P-p38MAPK from inflammatory nociception provokes the P-p38MAPK to increase faster during the formation of EA tolerance, which could explain that EA tolerance is induced faster in the inflammatory nociception model. Notably, our previous research revealed that IVC injection of let-7b-5p antagomir would not affect the TFL and PWT in rats without induction of EA tolerance. However, the same treatment would increase TFL and PWT in rats with induction of EA tolerance. There are two possible explanations. (1) There is a minimal requirement of P-p38MAPK expression in neuronal cells; let-7b-5p antagomir could not further decrease the P-p38MAPK level in rat without EA tolerance. (2) The baseline expression level of let-7b-5p is too low to be further reduced by antagomir. Therefore, the let-7b-5p antagomir could not exert any biological effects on its target gene. However, the exact biological mechanism of this phenomenon still demands further investigation.

MiRNAs achieve their biological function through post-transcriptional regulation of their target mRNAs. Previous report had found that miRNAs play vital role in the nociception in various nociception models. MiR-137, miR-96, miR-223, and miR103 take effects in neuropathic pain in either central or peripheral nervous tissues. However, their target is still not clear. Tao et al. reported that miR-199, miR-144, and miR-17 were able to affect visceral pain through interfering with nociception-related receptors or pathways including TRPV, Occludin, and STAT3. These results highlighted the modulatory effect of miRNA on nociception. Several miRNAs have been predicted to be involved in EA tolerance (Andersen et al. 2014; Tao et al. 2018). For example, miR-148a was reported to target the cholecystokinin type B receptor (CCKBR). This receptor interacts with the cholecystokinin octapeptide (CCK-8), which was reported to be implicated in EA tolerance through affecting the function of opioid receptor (Han 2001). In our previous study, we also found decreased miR-148a levels during the formation of EA tolerance. By ICV injection of its agomir or antagomir, we verified that miR-148a plays a role in EA tolerance by targeting the CCKBR. Other miRNAs, such as miR-124-3p, miR-434-5p, miR-107-3p, and let-7b-5p, have also been implicated in EA tolerance (Cui et al. 2017). Together with the result in this study, it showed that the EA tolerance is a complex biological process that may be regulated by multiple miRNAs.

The MKP-1 gene is a suppressor of the p38MAPK pathway and mainly works as a p38MAPK phosphatase (J. Julie et al. 2006). The activation of p38MAPK signal transduction pathway plays an important role in the development and maintenance of inflammatory nociception (Boyle et al. 2006; Sanjay et al. 2003), and EA could suppress the inflammatory nociception by inhibiting the same pathway (Fang et al. 2013). In this study, we found that let-7b-5p positively associated with the p38MAPK pathway. As the EA tolerance was formed and the level of let-7b-5p increased, the phosphorylation level of p38MAPK was also increased in the hypothalamus of CFA-induced inflammatory nociception rats. To further investigate the causal relationship between let-7b-5p and the MPK-1/p38MAPK pathway in the hypothalamus, let-7b-5p antagomir and agomir were injected intracerebroventricularly. As predicted, the P-p38MAPK level in the hypothalamus was significantly increased following injections of let-7b-5p agomir and was decreased when let-7b-5p antagomir was injected. In addition, the levels of MKP-1 were changed in the opposite direction of p38MAPK. Because MKP-1 is a negative regulator of P-p38MAPK and targeted by let-7b-5p, these results suggested that let-7b-5p could activate the phosphorylation of p38MAPK by downregulating MKP-1. As the activation of the p38MAPK pathway is a central mechanism for the analgesic effects of EA in the CFA-induced inflammatory nociceptive model, it is likely that let-7b-5p contributes to the formation of EA tolerance through activating the same pathway.

## Electronic supplementary material

ESM 1 (DOCX 16.3 kb)
