# Supplementary material for: MicroRNA Let-7b-5p Induces Electroacupuncture Tolerance by Downregulating the MKP-1 Gene in Rats Subjected to CFA-induced Inflammatory Nociception
Source: J Mol Neurosci. 2020 Apr 2;70(8):1198–207. doi: 10.1007/s12031-020-01527-6 (PMC7359146; doi:10.1007/s12031-020-01527-6)
Supplement: Supplementary file 1 — (DOCX 16.3 kb) [file 12031_2020_1527_MOESM1_ESM.docx]

**Supplementary Table 1**

**The partial results of the predicting target 3’UTR gene of let-7b-5p**

| Gene | RefseqID | Seed Length | Start | Sequence | End | Pvalue | SPMS |
| --- | --- | --- | --- | --- | --- | --- | --- |
| Cln3 | NM_001006971 | 7 | 1575 | GAGGUAG | 1569 | 0.0140 | 2 |
| Cln3 | NM_001006971 | 8 | 1576 | UGAGGUAG | 1569 | 0.0035 | 1 |
| Cmah | NM_001024273 | 7 | 1944 | GAGGUAG | 1938 | 0.0250 | 2 |
| Col1a1 | NM_053304 | 8 | 5251 | GAGGUAGU | 5244 | 0.0207 | 2 |
| Ctsb | NM_022597 | 7 | 1736 | UGAGGUA | 1730 | 0.0485 | 1 |
| Ctsf | NM_001034110 | 7 | 1536 | UGAGGUA | 1530 | 0.0302 | 1 |
| Cxcl2 | NM_053647 | 7 | 407 | UGAGGUA | 401 | 0.0435 | 1 |
| Cyb5d2 | NM_001007671 | 7 | 1170 | GAGGUAG | 1164 | 0.0291 | 2 |
| Cyp17a1 | NM_012753 | 7 | 1589 | UGAGGUA | 1583 | 0.0102 | 1 |
| Cyp2c22 | NM_138512 | 7 | 1617 | GAGGUAG | 1611 | 0.0132 | 2 |
| Cyp4f1 | NM_019623 | 7 | 1744 | GAGGUAG | 1738 | 0.0250 | 2 |
| Cyp8b1 | NM_031241 | 7 | 1609 | GAGGUAG | 1603 | 0.0260 | 2 |
| Dcakd | NM_001007724 | 8 | 1383 | GAGGUAGU | 1376 | 0.0128 | 2 |
| Ddx19_v2 | NM_001005381 | 8 | 1629 | UGAGGUAG | 1622 | 0.0159 | 1 |
| Defb24 | NM_001037508 | 7 | 363 | GAGGUAG | 357 | 0.0054 | 2 |
| Diablo | NM_001008292 | 7 | 955 | UGAGGUA | 949 | 0.0358 | 1 |
| Dkk3 | NM_138519 | 8 | 1551 | UGAGGUAG | 1544 | 0.0331 | 1 |
| Dph2 | NM_001015007 | 8 | 1907 | UGAGGUAG | 1900 | 0.0096 | 1 |
| Dph2 | NM_001015007 | 7 | 1906 | GAGGUAG | 1900 | 0.0380 | 2 |
| Duox2 | NM_024141 | 8 | 4700 | UGAGGUAG | 4693 | 0.0172 | 1 |
| *Dusp1* | NM_053769 | 7 | 1701 | UGAGGUA | 1695 | 0.0416 | 1 |
| Dzip1l | NM_001014095 | 8 | 3079 | GAGGUAGU | 3072 | 0.0177 | 2 |
| Efhd2 | NM_001031648 | 8 | 1320 | UGAGGUAG | 1313 | 0.0210 | 1 |
| Egln2 | NM_001004083 | 7 | 1980 | UGAGGUA | 1974 | 0.0298 | 1 |
| Eral1 | NM_001013229 | 7 | 1612 | GAGGUAG | 1606 | 0.0453 | 2 |
| Esam | NM_001004245 | 7 | 1396 | GAGGUAG | 1390 | 0.0339 | 2 |
| Fas | NM_139194 | 8 | 1040 | GAGGUAGU | 1033 | 0.0230 | 2 |
| Faslg | NM_012908 | 8 | 1258 | UGAGGUAG | 1251 | 0.0108 | 1 |
| Faslg | NM_012908 | 7 | 1257 | GAGGUAG | 1251 | 0.0426 | 2 |
| Fbxl12 | NM_001025700 | 8 | 1515 | UGAGGUAG | 1508 | 0.0097 | 1 |
| Fbxl12 | NM_001025700 | 7 | 1514 | GAGGUAG | 1508 | 0.0382 | 2 |
| Fbxo30 | NM_001007690 | 7 | 3156 | UGAGGUA | 3150 | 0.0497 | 1 |
| Fgfbp1 | NM_022603 | 7 | 945 | UGAGGUA | 939 | 0.0226 | 1 |
| Fmo1 | NM_012792 | 7 | 1759 | GAGGUAG | 1753 | 0.0241 | 2 |
| Frap1 | NM_019906 | 7 | 8139 | UGAGGUA | 8133 | 0.0500 | 1 |
| Gabbr2 | NM_031802 | 8 | 4243 | UGAGGUAG | 4236 | 0.0372 | 1 |
| Gabrd | NM_017289 | 7 | 1534 | UGAGGUA | 1528 | 0.0241 | 1 |
| Gale | NM_080783 | 8 | 1173 | UGAGGUAG | 1166 | 0.0053 | 1 |
| Gale | NM_080783 | 7 | 1172 | GAGGUAG | 1166 | 0.0209 | 2 |
| Galm | NM_001007704 | 8 | 1491 | UGAGGUAG | 1484 | 0.0170 | 1 |
